# The Joint Effect of Maternal Marital Status and Type of Household Cooking Fuel on Child Nutritional Status in Sub-Saharan Africa: Analysis of Cross-Sectional Surveys on Children from 31 Countries

**DOI:** 10.3390/nu13051541

**Published:** 2021-05-03

**Authors:** Iddrisu Amadu, Abdul-Aziz Seidu, Eric Duku, Joshua Okyere, John Elvis Hagan, Thomas Hormenu, Bright Opoku Ahinkorah

**Affiliations:** 1 Africa Centre of Excellence in Coastal Resilience, University of Cape Coast, Cape Coast PMB TF0494, Ghana; iddrisu.amadu@stu.ucc.edu.gh (I.A.); eric.duku@stu.ucc.edu.gh (E.D.); 2 Department of Fisheries and Aquatic Sciences, College of Agriculture and Natural Sciences, School of Biological Sciences, University of Cape Coast, Cape Coast PMB TF0494, Ghana; 3 Department of Population and Health, College of Humanities and Legal Studies, University of Cape Coast, Cape Coast PMB TF0494, Ghana; abdul-aziz.seidu@stu.ucc.edu.gh (A.-A.S.); joshuaokyere54@gmail.com (J.O.); 4 College of Public Health, Medical and Veterinary Sciences, James Cook University, Townsville, QLD 4811, Australia; thormenu@ucc.edu.gh; 5 Department of Health, Physical Education, and Recreation, University of Cape Coast, Cape Coast PMB TF0494, Ghana; 6 Neurocognition and Action-Biomechanics-Research Group, Faculty of Psychology and Sport Sciences, Bielefeld University, Postfach 10 01 31, 33501 Bielefeld, Germany; 7 School of Public Health, Faculty of Health, University of Technology Sydney, Sydney, NSW 2007, Australia; brightahinkorah@gmail.com

**Keywords:** biomass fuel, child nutrition, single-motherhood, solar, sub-Saharan Africa

## Abstract

The current study sought to investigate the joint effect of maternal marital status and type of household cooking fuel on child nutritional status in sub-Saharan Africa. Data in the children’s files of 31 sub-Saharan African countries were pooled from the Demographic and Health Surveys collected between 2010 and 2019. The outcome variables were three child anthropometrics: stunting (height-for-age z-scores); wasting (weight-for-height z-scores); and underweight (weight-for-age z-scores). The joint effect of maternal marital status and type of household cooking fuel on child nutritional status was examined using multilevel regression models. The results were presented as adjusted odds ratios (aORs) at *p* < 0.05. The percentages of children who were stunted, wasted and underweight in the 31 countries in sub-Saharan Africa were 31%, 8% and 17%, respectively. On the joint effect of maternal marital status and type of household cooking fuel on stunting, we found that compared to children born to married mothers who used clean household cooking fuel, children born to single mothers who use unclean household cooking fuel, children born to single women who use clean household cooking fuel, and children born to married women who used unclean household cooking were more likely to be stunted. With wasting, children born to single mothers who used unclean household cooking fuel and children born to married women who used unclean household cooking fuel were more likely to be wasted compared to children born to married mothers who used clean household cooking fuel. With underweight, we found that compared to children born to married mothers who used clean household cooking fuel, children born to single mothers who used unclean household cooking fuel, children born to single women who used clean household cooking fuel and children born to married women who used unclean household cooking were more likely to be underweight. It is imperative for the governments of the 31 sub-Saharan African countries to double their efforts to end the use of unclean household cooking fuel. This goal could be achieved by promoting clean household cooking fuel (e.g., electricity, gas, ethanol, solar, etc.) through effective health education, and promotion programmes. The attention of policymakers is drawn to the urgent need for children’s nutritional status policies and programmes (e.g., dietary supplementation, increasing dietary diversity, improving agriculture and food security) to be targeted towards at-risk sub-populations (i.e., single mothered households).

## 1. Introduction

Children are expected to receive the required nutritional needs to promote their physical and cognitive development [1]. Hence, child nutrition can be thought of as a fundamental right of children, with several countries across the globe contributing significantly towards the improvement of children’s nutritional status (CNS) [1]. Notwithstanding these efforts to reduce child morbidity and mortality, CNS (manifesting as stunting, wasting, or underweight) remains an obdurate public health concern, especially in sub-Saharan Africa (SSA) [2]. Reports from UNICEF [3] indicate that SSA recorded the highest prevalence of stunting (40%) and the second-highest prevalence of wasting (9%) worldwide in 2018. This is a worrying development for SSA, and therefore calls for urgent research to explore the underlying factors that result in the nutritional status of children within the region.

Prioritizing CNS is integral to the health, and wellbeing of the child sincepoor CNS may have deleterious repercussions (short and long term) on the individual. For instance, some studies have revealed that stunted children are at higher risk of experiencing poorer health, lower economic status, poor cognition, as well as lower educational performance [4,5].

Existing evidence on CNS has largely focused on how characteristics such as the age of the mother, poverty, and feeding practices [6,7,8] relate to CNS, with little attention given to the potential relationship between maternal marital status and CNS. However, available evidence suggests that there have been significant changes in the family structure, with an increasing incidence of out-of-wedlock motherhood, divorce, and widowhood being reported in SSA [9]. The effects of globalization, urbanization, and the HIV pandemic have been cited as potential reasons, hence resulting in an increased proportion of single mothers within the sub-region [9,10]. This proliferation of single mothers also arouses concerns that a substantial proportion of children born are being raised in a single-mother household [2].

Beyond the postulation that there may be an association between maternal marital status and CNS, there is a growing interest within the public health discipline concerning the role or association between household cooking fuel type and CNS [11,12,13,14]. Previous studies have demonstrated the association between cooking fuel and children’s health. For instance, in a study conducted by Owili et al. [11], it was found that the odds of an under-five child dying was higher for those whose households used charcoal and biomass cooking fuel compared to those who used clean fuel. Thus, a lack of access to and non-use of clean cooking fuel exacerbates under-five mortality. Other studies have also shown that under-fives may die when exposed to solid fuel usually used in the household within the sub-Saharan African context [15].

After an extensive literature search, we found no study in SSA that has explicitly examined the joint effect of maternal marital status and household cooking fuel type on CNS. Therefore, in the present study, we sought to contribute towards bridging the gap in the literature by investigating the joint effect of maternal marital status and householdcooking fuel type on CNSin SSA.

Our study is timely and significant in facilitating SSA’s quest to achieve the Sustainable Development Goals (SDG), particularly SDG 2.2, which envisions to end all forms of malnutrition by 2030, and also ensure that by 2025, the internationally agreed targets on stunting and wasting in children under five years of age are achieved [3].

## 2. Materials and Methods

### 2.1. Data Source

**Figure 1:**
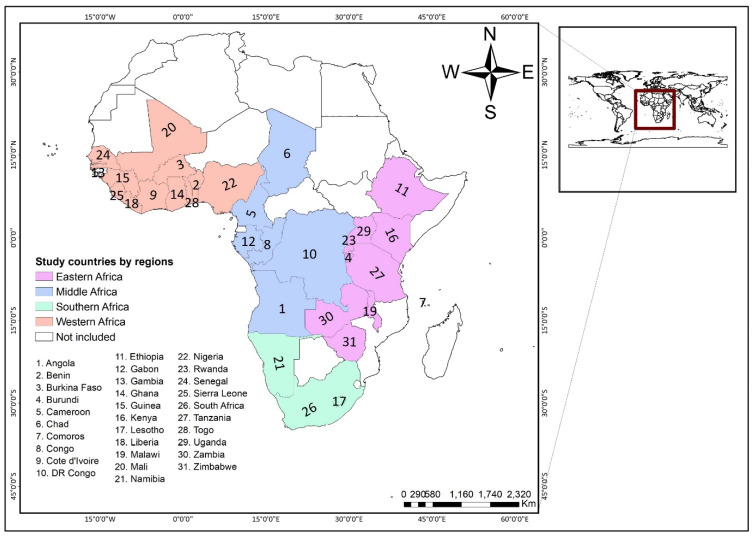
Map showing the 31 sub-Saharan African Countries.

Data for this study were obtained from the Demographic and Health Surveys (DHS) of 31 countries in SSA counducted from 2010 to 2019. The DHS Program has since 1984 assisted in the conduct of over 400 surveys in many low-and middle-income countries around the world. These cross-sectional surveys provide nationally representative household data on various nutrition, population and health indicators in more than 90 countries. Standardized protocols and instruments are employed to gather data of children, women, men and households. For this study, data in the children’s files were pooled from the DHS. The surveys employ a two-stage stratified sampling in selecting participants. The first stage involves the selection of clusters, usually called enumeration areas (EAs), and the second stage consists of the selection of households for the survey. To ensure consistency in data collection across countries, the DHS use a standard questionnaire comparable across countries for data collection, and the questionnaire is often translated into the major local languages of the countries involved. To ensure validity of the translated questionnaires, the DHS reports that the translated questionnaires, together with the version in English, are pretested in English and the local dialect [16,17]. Figure 1 shows the countries included in this study. We followed the Strengthening the Reporting of Observational Studies in Epidemiology’ (STROBE) statement in writing the manuscript. The dataset is freely accessible for download at: https://dhsprogram.com/data/available-datasets.cfm (accessed on 3 February 2021).

### 2.2. Measures

#### 2.2.1. Outcome Variables

The outcome variables are three child anthropometrics: stunting (height-for-age z-scores); wasting (weight-for-height z-scores); and underweight (weight-for-age z-scores). These variables were defined and coded using the WHO child growth standard which is followed by the DHS program [18]. The coding was done as follows:

- Stunting: children with height-for-age z-scores below minus 2 (−2.0) standard deviations less than the mean on the WHO Child Growth Standards (moderately or severely stunted) and children with height-for-age z-scores below minus 3 (−3.0) standard deviations less than the mean on the WHO Child Growth Standards (severely stunted) were combined to form the response group “Stunt” while those height-for-age z-scores equal to or higher than minus 2 (−2.0) standard deviations greater than the mean on the WHO Child Growth Standards were regarded as “not a stunt”.
- Wasting: children with weight-for-height z-scores below minus 2 (−2.0) standard deviations less than the mean on the WHO Child Growth Standards (moderately or severely wasting) and children with weight-for-height z-scores below minus 3 (−3.0) standard deviations less than the mean on the WHO Child Growth Standards (severely wasting) were combined to form the response group “Wasting” while those weight-for-height z-scores equal to or higher than minus 2 (−2.0) standard deviations greater than the mean on the WHO Child Growth Standards were regarded as “No wasting”.
- Underweight: children with weight-for-age z-scores below minus 2 (−2.0) standard deviations less than the mean on the WHO Child Growth Standards (moderately or severely underweight) and children with weight-for-age z-scores below minus 3 (−3.0) standard deviations less than the mean on the WHO Child Growth Standards (severely underweight) were combined to form the response group “Underweight” while those weight-for-age z-scores equal to or higher than minus 2 (−2.0) standard deviations greater than the mean on the WHO Child Growth Standards were regarded as “Not underweight”. For each of these variables, “age out of plausible limits”, “height out of plausible limits”, missing and “flagged” responses which constituted, 13, 744, 1833 and 3285 respectively were deemed invalid and dropped.

#### 2.2.2. Key Predictor Variable

The main predictor variables used were generated based on literature and potential contextual implications of findings. They were maternal marital status and type of household cooking fuel. The variable “maternal marital status” was coded to produce two responses as follows: never married, widowed and separated/divorced were coded together as “Single” and married and living with a partner as “Married” [9]. For parsimony, theoretical and contextual relevance, the variable “type of household cooking fuel” was also coded into two response categories “Clean” and “Unclean” following previous studies [19,20]. Clean fuels included electricity, liquefied petroleum gas (LPG) and natural gas while charcoal, firewood, grass/straw, dung, shrubs, agricultural crop waste represented unclean cooking fuels [19,20]. The two variables “maternal marital status” and “type of household cooking fuel” were then combined [19,20] to produce the variable “Maternal marital status-Type of cooking fuel” with four (4) mutually exclusive categories: “Single mother-clean” (single mothers living in a household that uses clean cooking fuel), “Single mother-unclean” (single mothers living in households that uses unclean cooking fuel), “Married -clean” (mothers who are married or living with a partner in a household that uses clean cooking fuel” and “Married -unclean” (mothers who are married or living with a partner in a household that uses unclean cooking fuel). To observe the effect of maternal marital status and the type of household cooking fuel on the nutritional status of children under the age of 5 years, married-clean is used as the reference group.

#### 2.2.3. Covariates

In the analysis of the effect of maternal marital status and household cooking fuel type on the nutritional status of children under age 5, three categories/clusters of variables (individual level factors-child and mother’s characteristics, household characteristics, and contextual factors) were considered ascovariates. The selection of these variables was based on their significant associations with CNS in previous studies (6–8). Variables under individual level factorsconsidered include the age of the child (0, 1, 2 and 4); sex of child (female and male); birth order of child (1, 2 to 4, and 5 and above); and perceived size at birth (small, average and large) (see [21]). Other included maternal age (re-coded into two categories “15–19” years and “20–49” years (see [22]); educational attainment (no formal education, primary, secondary and higher); working status (yes and no); antenatal visits during pregnancy (yes, no, and “Don’t know”); postnatal check within 2 months (yes and no); and place of delivery (home, health facility, other). At the household level, relevant variables included wealth status (recode as “poor”, “middle” and “rich”); the age of household head (recoded as ages below 35 years “young adults”, between 35 and 55 years “middle-aged adults” and those above 55 years “old-aged adults”; sex of household head (male and female); access to electricity (yes and no); type of toilet facility (re-coded into “improved” and unimproved”; source of drinking water (re-coded as “improved” and “unimproved” (see [23]); and access to media (yes, no) which was derived from the three variables “access to television”, “radio” and “newspaper/magazine”. The contextual factors considered are Urbanicity (rural and urban) and geographic region. The variable “Country” was re-coded to generate “Geographic region” following the UN’s list of countries and geographic regions in SSA.

### 2.3. Data Analyses

Stata SE version 14.2 (StataCorp, College Station, TX, USA) was used for statistical analyses of data. Descriptive statistics, including frequencies, percentages (weighted) and 95% confidence intervals (CIs) of percentages at *p* < 0.05 were used to summarize and present the data in tables. To enhance visualization and appreciation of the distributions of the outcome variables across the study countries, the data was integrated into a GIS environment and presented in map images. This procedure was then followed with a bivariate chi-square test of independence to determine the associations between the outcome variables and each of the key predictor variables and covariates. Collinearity diagnosis tests, including Variance Inflation Factors (VIF), Square VIF, Tolerance and R-squared were conducted for the key predictor variables and covariates. The joint effect of maternal marital status and type of household cooking fuel on CNS was examined using six multilevel regression models for each of the outcome variables (stunting, wasting, and underweight). The first model (Model 0) showed the variance in nutritional status attributed to the clustering of the primary sampling units (PSUs), without the explanatory variables. Model I contained only the key predictor variable (maternal marital status-type of household cooking fuel). Model II and III controlled for the individual and household level factors, respectively, while Model IV controlled for the contextual level factors. The final model (Model V) controlled for all the the individual, household, and contextual level factors. The Stata command “melogit” was used in fitting these models. We used Akaike’s Information Criterion (AIC) tests for Model comparison. All the results were presented using adjusted odds ratios (aOR) at 95% Confidence Interval (CI). To prevent potential challenges of oversampling or under-sampling and clustering of samples emerging from the multi-stage sampling technique used in the data collection, the weighting, cluster and strata variables were used to adjust the effect sizes.

### 2.4. Ethical Approval

For DHS reports, ethical clearance are sought from the Ethics Committee of ORC Macro Inc. as well as Ethics Boards of partner institutions (e.g., Ministries of Health) of the studied countries. The DHS protocols ensure that standards for the protection of respondents’ privacy and confidentiality are adhered. Inner City Fund International also make sure that the survey conforms with the United States Department of Health and Human Services’ regulations for the respect of human subjects. This study used a secondary data, hence, no further ethical approval was required. The datasets are freely available for download in the public domain. Further information about the DHS data usage and ethical standards is available at http://goo.gl/ny8T6X (accessed on 3 February 2021).

## 3. Results

### 3.1. Descriptive Analysis on the Percentage of Children Who Were Stunted, Wasted and Underweight in the 31 Countries in SSA

The study included 129,646 children under five from 31 sub-Saharan African countries. The percentage of children who were stunted, wasted, and underweight in the 31 countries in SSA considered was 31%, 8% and 17%, respectively (see Table 1).

**Figure 2:**
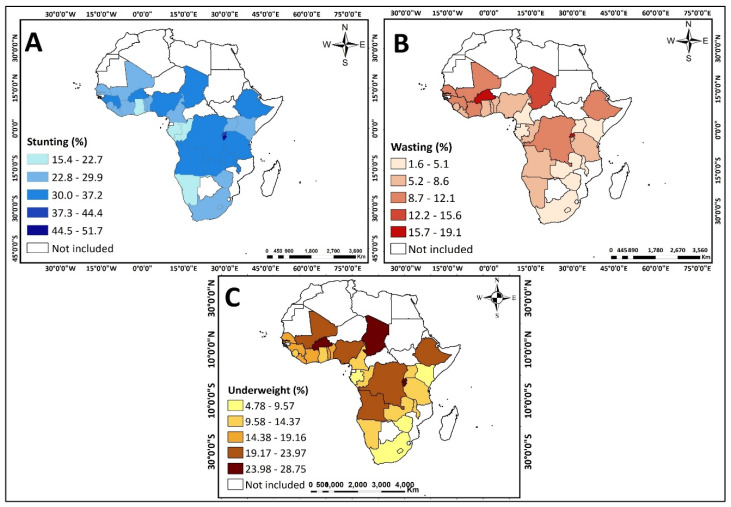
Maps showing the prevalence of stunting, wasting and underweight from 2010 to 2019 DHS across the 31 Sub-Saharan African Countries. (**A**) Stunting (%) (**B**) Wasting (%) (**C**) Underweight (%).

**Figure 3:**
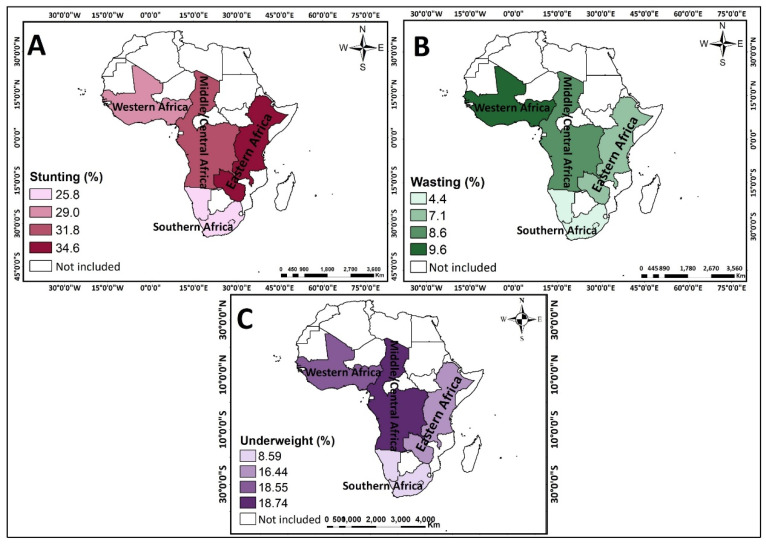
Maps showing the prevalence of stunting, wasting and undernutrition from 2010 to 2019 DHS across the 31 Sub-Saharan African Countries. (**A**) Stunting (%) (**B**) Wasting (%) (**C**) Underweight (%).

The prevalence of stunting varied across countries and sub-regions, with the highest prevalence of stunting found in Burundi (44.5%–51.7%), while the lowest prevalence was found in Congo, Gabon, Namibia, and Ghana (15.4%–22.7%) (see Figure 2A). In terms of sub-region, the highest prevalence of wasting was observed in Eastern Africa (34.6%) (see Figure 3A). The prevalence of wasting also varied across countries and sub-regions. The highest prevalence of wasting was found in Burundi, Burkina Faso and Chad (15.7%–19.1%), whereas the lowest prevalence was seen in South Africa, Lesotho, Zimbabwe, Zambia, Malawi, Kenya, Uganda, Rwanda, Gabon, Congo, and Cameroon (1.6%–5.1%) (see Figure 2B). Across sub-regions, wasting was more predominant in Western Africa (9.6%) (see Figure 2B). The highest prevalence of underweight was found in Burundi, Burkina Faso and Chad (24.0%–28.8%) (see Figure 2C) while the lowest prevalence was noted in South Africa, Lesotho, Zimbabwe, Kenya, and Gabon (4.8%–9.6%) (see Figure 2C).

Central/Middle Africa had the highest prevalence of underweight children across sub-regions (18.74%) (see Figure 3C).

In terms of the association between the key predictor variables, covariates and stunting, wasting, and underweight, we found significant associations between all the key predictor variables, covariates and stunting, except thematernal marital status and maternal age. Apart from age of the household head, all the independent variables had significant associations with wasting. With underweight, all the independent variables had significant associations, except the maternal age(see Table 2).

### 3.2. Multivariate Analysis on the Joint Effect of Single-Motherhood and Unclean Household Cooking Fuel Use on Child Nutritional Status

Table 3, Table 4 and Table 5 show the results from the multilevel logistic regression analysis on the joint effect of maternal marital status and household cooking fuel type on CNS. The last models (Model V) of each table indicate the joint effect of maternal marital status and household cooking fuel type on CNS, while controlling for individual level factors, household characteristics, and contextual factors. On the joint effect of maternal marital status and type of household cooking fuel on stunting, we found that compared to children born to married mothers who use clean household cooking fuel, children born to single mothers who use unclean household cooking fuel (aOR = 1.27; 95% CI = 1.17–1.47), children born to single women who use clean household cooking fuel (aOR = 1.18; 95% CI = 1.05–1.32) and children born to married women who use unclean household cooking (aOR = 1.25; 95% CI = 1.17–1.33) were more likely to be stunted (Table 3, Model V). With wasting, children born to single mothers who used unclean household cooking fuel (aOR = 1.17; 95% CI = 1.03–1.33) and children born to married women who use unclean household cooking fuel (aOR = 1.24; 95% CI = 1.11–1.39) were more likely to be wasted compared to children born to married mothers who used clean household cooking fuel (Table 4, Model V). With underweight, we found that compared to children born to married mothers who use clean household cooking fuel, children born to single mothers who use unclean household cooking fuel (aOR = 1.41; 95% CI = 1.28–1.55), children born to single women who use clean household cooking fuel (aOR = 1.33; 95% CI = 1.14–1.55) and children born to married women who use unclean household cooking (aOR = 1.33; 95% CI = 1.22–1.45) were more likely to be underweight (Table 3, Model V).

## 4. Discussion

Improving the health status of children has become an important global health issue. International organisations and individual countries have concentrated efforts and strategies to improve child nutritional status [1]. Yet, there are still a substantial proportion of children who are stunted, underweight or wasted [3]. Therefore, this study aimed to examine the joint effect of maternal marital status and household cooking fuel type on CNS in SSA.

The results indicated that there were some significant inter-country variations concerning the nutritional status of children. Consistently, our findings showed that Burundi had the worst children’s nutritional status (stunting, wasting, and underweight). This indicates that the indicators of child nutritional status in Burundi were poorer than those of other countries in SSA. Previous studies have found a high prevalence of stunting, wasting, and underweight among children in Burundi [24,25,26]. Besides Burundi, the prevalence of wasting and underweight was highest in Burkina Faso and Chad. This finding is in line with previous evidence that shows that wasting and underweight are most prevalent in Burkina Faso [27] and Chad [28].

We found that the prevalence of stunting is lowest in Ghana. This finding is consistent with previous outcomes in the country [6]. The identified pattern could be explained by the various strategies adopted by the government of Ghana to alleviate poverty and ensure food security for households to meet the children’s nutritional requirements. Notable among these interventions are the capitation grant and the school feeding programme, as well as the Livelihoods Empowerment Against Poverty Programme (LEAP) [29].

Concerning the joint effect of maternal marital status and type of household cooking fuel use on stunting, we found that compared to children born to married mothers who use clean household cooking fuel, children born to single mothers who use unclean household cooking fuel, single mothers who use clean household cooking fuel, and married mothers who use unclean household cooking fuel were more likely to be stunted. This finding corroborates recent studies (e.g., [30]) that the likelihood of childhood stunting among children living in households using unclean cooking fuel was significantly higher than those living in households using clean cooking fuel. For example, Balietti and [31] found increased odds of stunting among children whose household used unclean cooking fuel. Unclean cooking fuel coupled with poor ventilation increases household air pollution (HAP), which exacerbates the likelihood of stunting among children [32]. These result in respiratory infections that lead to the activation of the immune system to fight off disease-causing agents and consume metabolic energy, which will no longer be available for other functions of the metabolism. Child growth can be impaired, leading to stunting [33]. The association between use of unclean cooking fuel and stunting could also be explained from the perspective that, during pregnancy, mothers who used biofuel in the form of wood and dung have a higher risk of delivering small-for-gestational-age infants, with this outcome persisting during the growth of the child, and later causing stunting [34]. As found in this study, the use of unclean household cooking fuel influences stunting, together with single motherhood. Hence, children borrn to single mothers are also more likely to be stunted, irrespective of the type of household cooking fuel used by their mothers. The findings on the association between single motherhood and stunting is supported by previous studies [35,36,37].

Other findings also showed that children born to single mothers who used unclean household cooking fuel, single mothers who use clean household cooking fuel and married mothers who use unclean household cooking fuel were more likely to be underweight compared to children born to married mothers who used clean household cooking fuel. Thus, being a child born to a single mother who uses unclean household cooking fuel, a married mother who use unclean household cooking fuel and single mother who uses a clean household cooking fuel increases your likelihood of being underweight [12,13,14]. Generally, single mothers face substantial socio-economic stress and hardship in many parts of SSA [38]. These challenges limit the capacity of single mothers to provide quality child care for their children compared to married women [39]. As such, single mothers usually find it difficult to juggle between work and providing the nutritional needs of their children, hence, the higher likelihood of underweight among children born to single mothered households. The result is further supported by [40] which shows that the use of clean cooking fuel significantly reduces childhood underweight and mortality by 14–31%.

We also found that, compared to children born to married mothers who use clean household cooking fuel, those born to single and married mothers who use unclean household cooking fuel are more likely to be wasted. Hence, the use of unclean household fuel has significant effect on childhood stunting, irrespective of marital status. Similar finfings are evidenced in prior studies [12,41]. The effect of unclean cooking fuel and wasting could be attributed to the higher likelihood of delivering small-for-gestational-age infants among mothers who use unclean household cooking fuel, as this later can cause wasting [34].

### 4.1. Strength and Limitations

The study has several strengths. First, the use of a valid survey and rigorous statistical methods emphasise the trustworthiness and robustness of our findings. Additonally, the use of nationally representative data ensures that our findings are generalizable and replicable in the studied 31 sub-Saharan African countries. Moreover, current findings contribute to bridging the gaps in child nutrition literature which previous studies did not consider. Notwithstanding these strengths, the study has some limitations. Therefore, interpretations and inferences made from our findings must be considered in light of these limitations.

First, childhood nutritional status, maternal marital status and use of unclean cooking fuel interact in a complex system in SSA influenced by cultural beliefs. However, because the study relied on secondary data, the analysis was limited to only variables that were in the datasets. Further, the DHS employs cross-sectional designs, hence causal effects cannot be ascribed to the noted outcomes. However, only associative effects can be ascribed to the noted outcomes. The key predictor variables were self-reported by the mothers, and therefore, responses given are subject to recall biases and other social desirability concerns. Relatedly, self-reported cooking fuel type is only a crude proxy for exposure to household air pollution and, therefore, may not completely capture the relationship between exposure to particulate matter or carbon monoxide and health outcomes. Finally, the pooling of the data may be affected by heterogeneity across countries or regions.

### 4.2. Practical Implications

Practically, these findings demonstrate the need to invest in clean household cooking fuel. It is imperative for the governments of the 31 sub-Saharan African countries to double their efforts to end the use of unclean household cooking fuel. This could be achieved by going beyond just increasing access and affordability of clean cooking fuels (e.g., electricity, gas, ethanol, solar) by promoting their use through effective health education and health promotion programmes. Findings also draw the attention of policymakers to the urgent need for CNS policies and programmes (e.g., dietary supplementation, increasing dietary diversity, improving agriculture and food security) to be targeted towards at-risk sub-populations (i.e., single mothered households). Given the sub-regional variations noted in the current study, appropriate context-specific interventions are required to address household cooking fuel challenges and issues related to stunting, wasting and underweight among children in the studied 31 sub-Saharan African countries. 

## 5. Conclusions

The current study sought to examine the joint effect of maternal marital status and household cooking fuel type on child nutritional status in SSA. We found that maternal marital status and household cooking fuel type have a joint effect on childhood stunting, wasting and underweight. Future studies could explore the cultural dimension to this association between being a single mother, the use of unclean cooking fuel, and child nutritional status in SSA. Undertaking such studies would be instrumental in informing appropriate culturally sensitive strategies that can effectively improve CNS in SSA.

## Figures and Tables

**Table 1:** Distribution of study variables from 2010 to 2019 DHS data of 31 sub-Saharan African countries.

Variable	Weighted N	Weighted %	Variable	Weighted N	Weighted %
**Key outcome variables**			**Household characteristics**		
Stunting	40,453	31	Wealth status		
Wasting	10,770	8	Poor	54,734	42
Underweight	22,503	17	Middle	26,262	20
			Rich	48,650	38
**Key predictor variable**			Age of household head		
Maternal marital status			Young-adults	55,803	43
Single	14,071	11	Middle-aged adults	56,748	44
Married	2645	2	Old-aged adults	17,094	13
Type of cooking fuel	102,192	79	Sex of household head		
Clean Unclean	10,725	8	Male	103,254	80
**Child characteristics**			Female	26,392	20
Age			Access to electricity		
0	39,906	31	No	89,364	69
1	36,828	28	Yes	40,272	31
2	26,214	20	Type of toilet facility		
3	16,128	12	Improved	55,247	43
4	10,571	8	Unimproved	74,375	57
Sex			Source of drinking water		
Male	65,442	50	Improved	85,103	66
Female	64,204	50	Unimproved	44,531	34
Birth order			Type of cooking fuel		
1	25,421	20	Unclean	116,263	90
2 to 4	62,426	48	Clean	13,369	10
5 and above	41,799	32	Access to media (tv/radio/newspaper)		
Perceived size at birth			No	45,684	35
Large	43,855	34	Yes	83,962	65
Average	64,360	50	**Contextual factors**		
Small	21,422	17	Urbanicity		
Weight at birth			Urban	43,412	33
Underweight	7180	6	Rural	86,234	67
Normal	72,089	56	Country		
Not taken	50,377	39	Angola	7384	6
Stunting			Benin	15,857	12
Severely/moderately stunting	40,454	31	Burkina Faso	8908	7
No stunting	89,192	69	Burundi	4174	3
Wasting			Cameroon	2936	2
Severely/moderately wasting	10,770	8	Chad	6114	5
No wasting	118,876	92	Comoros	1523	1
Underweight			Congo	2620	2
Not underweight	107,136.99	83	Cote d’Ivoire	2190	2
Underweight	22,503.33	17	DR Congo	4842	4
**Mother’s characteristics**			Ethiopia	6585	5
Maternal marital status			Gabon	1998	2
single	16,718	13	Gambia	746	1
Married	112,928	87	Ghana	1908	1
Maternal Age			Guinea	2291	2
15–19	9230	7	Kenya	6082	5
20–49	120,416	93	Lesotho	1018	1
Educational attainment			Liberia	2035	2
No education	54,150	42	Malawi	4035	3
Primary	40,339	31	Mali	5785	4
Secondary	31,071	24	Namibia	1155	1
Higher	4086	3	Nigeria	7502	6
Working status			Rwanda	4424	3
No	46,034	36	Senegal	1989	2
Yes	83,505	64	Siera Leone	3052	2
Antenatal visits during pregnancy			South Africa	877	1
No	13,711	11	Tanzania	6088	5
Yes	113,765	88	Togo	2227	2
Dont’ know	2153	2	Uganda	2900	2
Postnatal check within 2 months			Zambia	6402	5
No	75,893	59	Zimbabwe	4000	3
Yes	53,753	41	Geographic region		
Place of delivery			Western Africa	54,489	42
Home	40,300	31	Eastern Africa	42,213	33
Health facility	87,846	68	Central Africa	25,894	20
Other	1494	1	South Africa	7051	5
			**Total**	**129,646**	

**Table 2:** Association between stunting, wasting and underweight and characteristics of a child, mother and household, and contextual factors from 2010 to 2019 DHS across the 31 Sub-Saharan African Countries.

Independent Variables	Stunting (Weighted %)	95% CI	*p*-Value	Wasting (Weighted %)	95% CI	*p*-Value	Underweight (Weighted %)	95% CI	*p*-Value
**Key predictor variables**									
**Maternal marital status**			0.382			<0.001			<0.001
Single	31.3	30.5–32.0		6.4	6.0–6.7		15.5	15.0–16.1	
Married	31.2	30.9–31.5		8.6	8.4–8.8		17.6	17.4–17.9	
**Type of cooking fuel**			<0.001			<0.001			<0.001
Unclean	32.6	32.4–32.9		8.7	8.5–8.9		18.3	18.1–18.5	
Clean	18.8	18.1–18.4		5.0	4.6–5.3		9	8.5–9.5	
**Child characteristics**									
**Age of child**			<0.001			<0.001			<0.001
0	16.8	16.4–17.2		11.1	10.8–11.4		13.56	13.2–13.0	
1	36.3	35.8–36.8		9.7	9.4–10.0		19.97	19.6–20.4	
2	43.5	42.9–44.1		6.2	5.9–6.5		20.59	20.1–21.1	
3	37.2	36.438.0		4.3	4.0–4.6		16.84	16.3–17.4	
4	28.2	27.3–29.0		4.4	4.0–4.8		15.35	14.7–16.1	
**Sex of child**			<0.001			<0.001			<0.001
Male	34.0	33.6–34.4		9.2	8.9–9.4		18.84	18.5–19.1	
Female	28.3	28.0–28.7		7.5	7.2–7.7		15.85	15.6–16.1	
**Birth order**			<0.001			<0.001			<0.001
1	29.5	28.9–30.0		7.5	7.2–7.8		15.23	14.8–15.7	
2 to 4	30.0	29.4–30.1		7.9	7.7–8.2		16.15	15.9–16.4	
5 and above	34.4	33.9–34.8		9.3	9.1–9.6		20.45	20.0–20.8	
**Perceived size at birth**			<0.001			<0.001			<0.001
Large	26.7	26.3–27.1		6.4	6.2–6.6		12.93	12.6–13.2	
Average	31.4	31.0–31.8		8.3	8.1–8.5		17.02	16.7–17.3	
Small	39.8	39.1–40.4		12.2	11.7–12.6		27.45	26.9–28.0	
**Mother’s characteristics**									
Maternal age			0.604			<0.001			0.189
15–19	31.0	30.0–31.9		9.6	9.0–10.2		17.68	16.9–18.5	
20–49	31.2	31.0–31.5		8.2	8.1–8.4		17.33	17.1–17.5	
**Educational attainment**			<0.001			<0.001			<0.001
No education	36.6	36.2–37.0		11.5	11.3–11.8		24.02	23.7–24.3	
Primary	32.6	32.1–33.0		6.4	6.2–6.7		15.1	14.8–15.5	
Secondary	22.6	22.2–23.1		5.6	5.3–5.9		10.19	9.9–10.5	
Higher	10.9	10.0–11.9		4.6	4.0–5.3		5.81	5.1–6.6	
**Working status**			0.017			<0.001			<0.001
No	30.8	30.3–31.2		9.6	9.4–9.9		18.16	17.8–18.5	
Yes	31.4	31.1–31.8		7.6	7.4–7.7		16.91	16.7–17.3	
**Antenatal visits during pregnancy**			<0.001			<0.001			<0.001
No	41.7	41.0–42.6		12.5	12.0–13.1		28.42	27.7–29.2	
Yes	30.0	29.8–30.3		7.8	7.7–8.0		16.1	15.9–16.3	
Dont’know	26.3	24.4–28.2		7.6	6.5–8.8		13.07	11.7–14.6	
**Postnatal check within 2 months**			<0.001			0.748			<0.001
No	33.0	32.6–33.3		8.3	8.1–8.5		18.54	18.3–18.8	
Yes	28.7	28.3–29.1		8.4	8.1–8.6		15.69	15.4–16.0	
**Place of delivery**			<0.001			<0.001			<0.001
Home	38.5	38.1–39.0		11.2	11.0–11.5		24.9	24.5–25.3	
Health facility	27.8	27.5–28.1		7.0	6.8–7.1		13.87	13.6–14.1	
Other	34.8	32.4–37.3		7.5	6.2–9.0		19.26	17.3–21.4	
**Household characteristics**									
**Wealth status**			<0.001			<0.001			<0.001
Poor	37.2	36.8–37.6		9.4	9.1–9.6		21.28	20.9–21.6	
Middle	32.6	32.0–33.2		8.2	7.9–8.6		17.8	17.3–18.3	
Rich	23.7	23.4–24.1		7.2	6.9–7.4		12.71	12.4–13.0	
**Age of household head**			0.680			0.791			<0.001
Young-adults	31.5	31.1–32.0		8.2	7.9–8.4		16.8	16.5–17.1	
Middle-aged adults	30.9	30.5–31.3		8.5	8.2–8.7		17.83	17.5–18.1	
Old-aged adults	31.1	30.4–31.8		8.3	7.9–8.7		17.61	17.0–18.2	
**Sex of household head**			<0.05			<0.001			<0.001
Male	31.4	31.1–31.7		8.7	8.5–8.9		17.78	17.5–18.0	
Female	30.5	30.0–31.0		6.9	6.6–7.3		15.72	15.3–16.2	
**Access to electricity**			<0.001			<0.001			<0.001
No	35.3	35.0–35.6		9.2	9.0–9.4		20.01	19.7–20.3	
Yes	22.1	21.7–22.5		6.3	6.0–6.5		11.47	11.1–11.8	
**Type of toilet facility**			<0.001			<0.001			<0.001
Improved	26.8	26.4–27.2		6.4	6.2–6.6		13.07	12.8–13.3	
Unimproved	34.5	34.1–34.8		9.7	9.5–9.9		20.54	20.3–20.8	
**Source of drinking water**			<0.001			<0.05			<0.001
Improved	30.1	29.8–30.4		8.2	8.0–8.4		16.64	16.4–16.9	
Unimproved	33.4	32.9–33.8		8.5	8.3–8.8		18.72	18.4–19.1	
**Access to media (tv/radio/newspaper)**			<0.001			<0.001			<0.001
No	37.9	37.4–38.3		10.0	9.7–10.2		22.43	22.0–22.8	
Yes	27.6	27.3–27.9		7.4	7.2–7.6		14.6	14.4–14.8	
**Contextual factors**									
**Urbanicity**			<0.001			<0.001			<0.001
Urban	23.6	23.2–24.0		6.6	6.3–6.8		12.12	11.8–12.4	
Rural	35.0	34.7–35.4		9.2	9.0–9.4		20	19.7–20.3	
**Geographic region**			<0.001			<0.001			<0.001
Western Africa	29.0	28.6–29.4		9.6	9.4–9.8		18.55	18.2–18.9	
Eastern Africa	34.6	34.1–35.0		7.1	6.9–7.4		16.44	16.1–16.8	
Central Africa	31.8	31.3–32.4		8.6	8.3–8.9		18.74	18.3–19.2	
South Africa	25.8	24.7–26.8		4.4	3.9–4.9		8.59	8.0–9.3	

**Table 3:** Multilevel logistic regression results on joint effect of maternal marital status and type of household cooking fuel on childhood stunting from 2010 to 2019 DHS across the 31 Sub-Saharan African Countries.

Key Predictor Variable	Model 0	Model I	Model II	Model III	Model IV	Model V
		OR (95% CI)	aOR (95% CI)	aOR (95% CI)	aOR (95% CI)	aOR (95% CI)
**Fixed effects**						
**Maternal marital status-Cooking fuel**						
Married-clean		1	1	1	1	1
Single unclean		2.22 *** (2.08–2.36)	1.65 *** (1.54–1.77)	1.49 *** (1.39–1.60)	1.83 *** (1.71–1.95)	1.27 *** (1.17–1.47)
Single clean		1.25 *** (1.12–1.39)	1.21 *** (1.08–1.36)	1.22 *** (1.09–1.36)	1.23 *** (1.10–1.37)	1.18 * (1.05–1.32)
Married-unclean		2.25 *** (2.03–2.26)	1.49 *** (1.41–1.59)	0.04 *** (1.32–1.48)	1.76 *** (1.66–1.87)	1.25 *** (1.17–1.33)
**Random effects**						
PSU Variance (95% CI)	0.02 (0.18–0.03)	0.02 (0.02–0.3)	0.02 (0.02–0.03)	0.02 (0.01–0.03)	0.02 (0.01–0.03)	0.02 (0.01–0.03)
ICC	0.007	0.007	0.006	0.006	0.006	0.006
LR Test	161.74 ***	141.62 ***	111.82 ***	118.11 ***	121.89 ***	105.02 ***
Wild χ2	Reference	864.24 ***	10,324.15 ***	2958.64 ***	2061.47 ***	11,264.12 ***
**Model fitness**						
Log-likelihood	−81,022.8	−80,532.1	−74,861.3	−79,409.4	−79,911.5	−74,213.7
AIC	162,049.6	161,074.3	149,770.6	158,846.9	159,841	148,501.4
Number of clusters	1608	1608	1608	1608	1608	1608

* *p* < 0.05; *** *p* < 0.001; Model 0: Empty model with no independent variables; Model I: Joint effect of maternal marital status and household cooking fuel type on stunting; Model II: Included individual level characteristics (age of child in years, sex of child, birth order and perceived size of child at birth, maternal age, educational attainment, working status, antenatal visits during pregnancy, postnatal check within 2 months and place of delivery) as covariates; Model III: Included household characteristics (wealth status, age of household head, sex of household head, access to electricity, type of toilet facility, source of drinking water and access to media) as covariates; Model IV: Included contextual factors (urbanicity and geographic region) as covariates; Model V: Included individual, household and contextual level characteristics as covariates Exponentiated coefficients; 95% confidence intervals in brackets; cOR: crude odds ratios; aOR adjusted odds ratios; CI Confidence Interval; 1 = Reference category; PSU = Primary Sampling Unit; ICC = Intra-Class Correlation; LR Test = Likelihood ratio Test; AIC = Akaike’s Information Criterion.

**Table 4:** Multilevel logistic regression results on joint effect of maternal marital status and type of household cooking fuel on childhood wasting from 2010 to 2019 DHS across the 31 Sub-Saharan African Countries.

Key Predictor Variable	Model 0	Model I	Model II	Model III	Model IV	Model V
		OR (95% CI)	aOR (95% CI)	aOR (95% CI)	aOR (95% CI)	aOR (95% CI)
**Fixed effects**						
**Maternal marital status-Cooking fuel**						
Married-clean		1	1	1	1	1
Single unclean		1.46 *** (1.30–1.63)	1.23 ** (1.09–1.39)	1.12 (0.99–1.27)	1.32 *** (1.17–1.49)	1.17 * (1.03–1.33)
Single clean		0.92 (0.74–1.14)	0.91 (0.73–1.13)	0.94 (0.75–1.17)	0.97 (0.78–1.20)	0.97 (0.78–1.21)
Married-unclean		1.98 *** (1.80–2.19)	1.35 *** (1.22–1.50)	1.45 *** (1.30–1.61)	1.69 *** (1.54–1.88)	1.24 *** (1.11–1.39)
**Random effects**						
PSU Variance (95% CI)	0.04 (0.03–0.06)	0.4 (0.03–0.06)	0.04 (0.03–0.05)	0.04 (0.03–0.05)	0.04 (0.03–0.5)	0.04 (0.03–0.5)
ICC	0.012	0.013	0.012	0.012	0.013	0.012
LR Test	84.12 ***	86.79 ***	78.58 ***	82.1 ***	86.54 ***	77.54 ***
Wild χ2	Reference	302.86 ***	3430.13 ***	813.45 ***	633.06 ***	3623.65 ***
**Model fitness**						
Log-likelihood	−37,598.7	−37,423.4	−35,695.6	−37,145.5	−37,243.6	−35,562.1
AIC	75,201.42	74,856.82	71,439.27	74,319.03	74,505.22	71,198.25
Number of clusters	1608	1608	1608	1608	1608	1608

* *p* < 0.05; ** *p* < 0.01; *** *p* < 0.001; Model 0: Empty model with no independent variables; Model I: Joint effect of maternal marital status and household cooking fuel type on wasting; Model II: Included individual level characteristics (age of child in years, sex of child, birth order and perceived size of child at birth, maternal age, educational attainment, working status, antenatal visits during pregnancy, postnatal check within 2 months and place of delivery) as covariates; Model III: Included household characteristics (wealth status, age of household head, sex of household head, access to electricity, type of toilet facility, source of drinking water and access to media) as covariates; Model IV: Included contextual factors (urbanicity and geographic region) as covariates; Model V: Included individual, household and contextual level characteristics as covariates; Exponentiated coefficients; 95% confidence intervals in brackets; cOR: crude odds ratios; aOR adjusted odds ratios; CI Confidence Interval; 1 = Reference category; PSU = Primary Sampling Unit; ICC = Intra-Class Correlation; LR Test = Likelihood ratio Test; AIC = Akaike’s Information Criterion.

**Table 5:** Multilevel logistic regression results on joint effect of maternal marital status and type of household cooking fuel on childhood underweight from 2010 to 2019 DHS across the 31 Sub-Saharan African Countries.

Key Predictor Variable	Model 0	Model I	Model II	Model III	Model IV	Model V
		OR (95% CI)	aOR (95% CI)	aOR (95% CI)	aOR (95% CI)	aOR (95% CI)
**Fixed effects**						
**Marital status-Cooking fuel**						
Married-clean		1	1	1	1	1
Single unclean		2.26 *** (2.08–2.47)	1.62 *** (1.48–1.77)	1.46 *** (1.32–1.60)	1.87 *** (1.71–2.04)	1.41 *** (1.28–1.55)
Single clean		1.21 *** (1.13–1.52)	1.28 * (1.10–1.50)	1.29 * (1.11–1.50)	1.38 *** (1.19–1.61)	1.33 *** (1.14–1.55)
Married-unclean		2.60 *** (2.41–2.80)	1.56 *** (1.44–1.69)	1.61 *** (1.48–1.74)	2.01 *** (1.86–2.18)	1.33 *** (1.22–1.45)
**Random effects**						
PSU Variance (95% CI)	0.03 (0.02–0.04)	0.03 (0.02–0.04)	0.02 (0.02–0.03)	0.03 (0.02–0.03)	0.03 (0.02–0.04)	0.02 (0.02–0.03)
ICC	0.009	0.009	0.007	0.008	0.008	0.007
LR Test	124.1 ***	118.46 ***	76.82 ***	101.51 ***	115.42 ***	80.61 ***
Wild χ2	Reference	707.76 ***	6725.19 ***	2423.9 ***	1821.05 ***	7154.32 ***
**Model fitness**						
Log-likelihood	−60,841.6	−60,404.4	−57,078.2	−59,474.5	−59,780.2	−56,749.8
AIC	121,687.2	120,818.7	114,204.4	118,977	119,578.4	113,573.6
Number of clusters	1608	1608	1608	1608	1608	1608

* *p* < 0.05; *** *p* < 0.001; Model 0: Empty model with no independent variables; Model I: Joint effect of maternal marital status and household cooking fuel type on underweight; Model II: Included individual level characteristics (age of child in years, sex of child, birth order and perceived size of child at birth, maternal age, educational attainment, working status, antenatal visits during pregnancy, postnatal check within 2 months and place of delivery) as covariates; Model III: Included household characteristics (wealth status, age of household head, sex of household head, access to electricity, type of toilet facility, source of drinking water and access to media) as covariates; Model IV: Included contextual factors (urbanicity and geographic region) as covariates; Model V: Included individual, household and contextual level characteristics as covariates; Exponentiated coefficients; 95% confidence intervals in brackets; cOR: crude odds ratios; aOR adjusted odds ratios; CI Confidence Interval; 1 = Reference category; PSU = Primary Sampling Unit; ICC = Intra-Class Correlation; LR Test = Likelihood ratio Test; AIC = Akaike’s Information Criterion.

## Data Availability

The dataset is available on the following website: http://goo.gl/ny8T6X (accessed on 3 February 2021).
